# Analysing root roughness and smear layer relationship by comparing contemporary dental curettes with conventional dental curettes: a randomised controlled trial

**DOI:** 10.1186/s12903-022-02268-1

**Published:** 2022-06-17

**Authors:** Sania Riaz, Shahbaz Ahmed, Sumaiya Shabbir, Ziaur Rahman Khan, Syed Jaffar Abbas Zaidi, Meshal Muhammad Naeem, Waqas Ahmed Farooqui

**Affiliations:** 1 Department of Periodontology, Baqai Dental College, Baqai Medical University, Karachi, Pakistan; 2 Department of Operative Dentistry, Dr Ishrat Ul Ebad Khan Institute of Oral Health Sciences, Dow University of Health Sciences, Karachi, Pakistan; 3 Department of Periodontology, Dow International Dental College, Dow University of Health Sciences, Karachi, Pakistan; 4 Department of Oral Medicine, Baqai Dental College, Baqai Medical University, Karachi, Pakistan; 5 Department of Oral Biology, Dow Dental College, Dow University of Health Sciences, Karachi, Pakistan; 6 Department of Periodontology, Dr Ishrat Ul Ebad Khan Institute of Oral Health Sciences, Dow University of Oral Health Sciences, Karachi, Pakistan; 7 School of Public Health, Dow University of Health Sciences, Karachi, Pakistan

**Keywords:** Scaling and root planning, Root roughness, Smear layer, Gracey curette, After-five curette, Mini-five curette

## Abstract

**Background:**

Root debridement procedures for the treatment of periodontal diseases, produces root surface irregularities and smear layer on the root surface that can adversely affect the healing of periodontal tissues. The objective of the present study was to evaluate the surface texture of root after hand instrumentation with Gracey curette, After Five curette, and Mini Five curette.

**Methods:**

A randomised, controlled clinical trial was conducted with 120 participants clinically diagnosed with chronic periodontitis. Participants were equally randomised into four groups, with parallel treatment assignment of scaling and root planning using Gracey Curettes, After five and Mini five curette, and a control group with no instrumentation. Mobile teeth of these patients were then extracted atraumatically and analysed under a Scanning Electron Microscope and graded for "Roughness and Loss of Tooth substance index" and "Smear layer index." Cross Tabulation was made between the test groups (Control, Gracey Curette, After five, and Mini Five) versus "Roughness and Loss of Tooth substance Index" and "Smear Layer Index." A Chi-square test with Bonferroni correction was used to determine the graded distribution among the groups.

**Results:**

In the control group, 73.3% of the teeth showed grade 1 roughness. In the Gracey group, 56.7% showed grade 2 roughness; in the After 5 group, 70% showed grade 3 roughness; in the Mini 5 group, 76.7% showed grade 3 roughness. A significant association was found between roughness scores and the use of individual instruments. Regarding smear layer formation, 46.7% of teeth showed a grade 4 smear layer in the control group. 50% of teeth showed grade 2 smear layer thickness in the Gracey group. In the After 5 group, 73.3% of teeth showed a grade 1 smear layer, while in the Mini 5 group, 80.0% showed a grade 1 smear layer. The use of individual instruments was significantly associated with the smear layer scores.

**Conclusion:**

Gracey curettes produced relatively smoother root surfaces with less smear layer formation than After Five and Mini Five curettes, which produced relatively more roughened root surfaces with thicker smear layer formation.

***Trial registration*:**

ID: ClinicalTrials.gov Identifier: NCT04216966 Date of Registration: January 3, 2020.

## Background

Root instrumentation is an integral component of surgical and non-surgical therapy of periodontitis that may leave root surfaces rough and irregular resulting in increased smear layer formation. However, periodontal healing is better in the absence of a smear layer [1]. An important goal of surface root roughness is to prevent the formation of new plaque deposits and to provide a biocompatible surface for the adhesion of periodontal fibroblasts, rather than bacteria. Therefore, selecting an optimal scaling hand instrument is vitally important as it can impact clinical outcomes such as clinical attachment loss, pocket depth, and bleeding upon probing.

A growing body of evidence suggests that plaque and calculus are the principal etiological agents of periodontal disease [2]. Roughened root surface accumulation favors bacterial attachment [3, 4]. The calculus is the ideal substrate for the colonisation of subgingival microbes [5]. Consequently, anti-infective treatment and root instrumentation aim to eliminate the cause by periodically mechanically debriding this diseased root surface [6].

An earlier belief was that bacterial endotoxins were tightly bound to root surfaces, so aggressive root planning was carried out [7]. The current evidence proposes that there is weak adherence of bacterial endotoxin to the root surface, so aggressive root planing and intentional removal of the infected cementum and root surface are not necessary to heal periodontal tissues [8]. Therefore, root instrumentation must be performed with instruments that remove minimal root substances and effectively disrupt the biofilm and remove calculus. Numerous studies have been conducted to determine which instruments lead to less root roughness [9]. While some believe that ultrasonic instruments are better at removing plaque and biofilm, others claim that hand instruments, such as curettes, are more effective [10]. The ideal treatment would provide effective debridement and preserves the tooth structure [11].

Clinically, the surface roughness of intraoral hard tissues plays an important role in retaining bacteria in biofilms of plaque. Calculus and biofilm are not simply organic deposits on the root surfaces, but they are embedded in the cemental irregularities, which act as a reservoir for endotoxins [12]. It has been established that increased roughness results in greater adhesion of bacteria and biofilm formation [13]. According to Bollen et al., an increase in surface roughness of these hard substances from 0.2 to 0.8 µm significantly affects plaque and biofilm formation. Therefore, an acceptable "Threshold Roughness" was proposed as "0.2 µm" [14].

A root surface that is diseased or has deposits of smear layer does not provide a favorable environment for cell attachment [15, 16]. During root-planing, the diseased root surfaces are covered by a smear layer made up of infected root cementum, toxins of bacteria, plaque, and calculus remnants [17–19].

The primary goal of scaling and root planing is to encourage the formation of a new epithelial attachment resulting from the migration of the periodontal ligament to the cleaned root surfaces. The smear layer interferes with the adhesion and formation of new attachments in most cases [20, 21]. Smear layers form after root debridement, causing a barrier between the root surface and periodontal cells, altering the healing process [22]. The goal of root treatment procedures is to make the root biologically compatible with the host tissues for optimal healing and better clinical outcomes [23].

According to Brannstorm, under Scanning Electron Microscopy (SEM), the smear layer appears to be amorphous, granular, and irregular [24]. Goldman et al^.^ [25] reported its thickness between 1 and 5 µm depending on the cutting instrument's sharpness and type [26]. For the fibrous reattachment to occur, the fibroblasts must contact the root surfaces; otherwise, a long junctional epithelium will form. Matheus Andre Muller investigated the effect of different Gracey curettes on the roughness of the root surfaces of teeth through SEM and found that the quality of the curette's cutting surface significantly affected root surface homogeneity [27]. Colette Landry compared Gracey curette with the Curvette Sub 0 curette to measure their effects on root roughness. Based on root surface microroughness, he concluded that Curvette Sub 0 type of curettes resulted in rougher roots [28].

A study by Richard J Nagy compared Extended Shanks curettes to the standard Gracey Curettes. This study evaluated the efficiency of curettes in terms of relative root roughness. The Gracey curette had a greater mean curette efficiency of 3.46 mm versus 2.77 mm [29].

Numerous studies have compared hand instruments with power-driven instruments and lasers for assessing root surface roughness [30, 31]. Based on different methodologies, these studies have produced variable and inconclusive results [32, 33]. While there is no universally accepted method for root planning and scaling, hand instruments produce smoother and cleaner results. To the best of our knowledge, no research has been conducted comparing conventional hand instruments such as the Gracey curette with contemporary hand instruments such as the After Five and Mini Five curettes.

The rationale of the present study is based on the fact that all forms of inflammatory gum diseases are managed by debridement, and therefore an essential aspect of periodontal therapy is the instrumentation of the root surfaces [34]. In periodontal treatment, the goal is to produce healthy and regenerating roots that are biologically adequate, acceptable, and smooth enough [35]. In clinical conditions, tooth substance loss is also affected by the shape and design of the instrument [36]. It is essential to remove as minimal as possible of the tooth structure because it can lead to serious complications in the future [37]. As a result, this trial was conducted due to limited and inconsistent evidence regarding the effectiveness of modified versions of curettes on root topography.

The present study compares the ultra-morphology of root surfaces induced by mechanical instrumentation by conventional Gracey curette and its modified version After Five curette, whose terminal shank is elongated by 3 mm for access in deep periodontal pockets and blade is 10% thinner. Mini Five curettes are those in which the terminal shank is elongated by 3 mm, and the blade is reduced to half-length of After Five or a standard Gracey curette, and the blade is 10% thinner than the standard Gracey curette. The objective of this study was to compare and evaluate the effects of Gracey, After Five, and mini-Five curettes on root surface roughness and deposition of the smear layer using scanning electron microscopy (SEM).

## Methods

### Study design and settings

A single-blinded randomised controlled trial was conducted at Dow University of Health Sciences, Karachi, Pakistan, one of the largest tertiary care dental hospitals in Pakistan, from March 7, 2018, to October 7, 2018. Ethical approval was sought before the commencement of the trial by the Ethical Review Committee of the Institutional Review Board, Dow University of Health Sciences [Ref. No.: IRB-952/DUHS/Approval/2017/173] dated December 9, 2017*.* The research protocol was registered with the Protocol Registration and Results System at ClinicalTrials.gov Identifier: NCT04216966 https://clinicaltrials.gov/ct2/show/NCT04216966 on 03/01/2020 following the CONSORT guidelines.

### Characteristics of participants

Each trial participant signed an informed written consent form before enrollment. Healthy Individuals were included who had single-rooted teeth planned for extraction and were diagnosed with severe chronic periodontitis having a clinical attachment loss of > 5 mm with Miller's class 3 mobility. The study excluded patients with teeth that had received root canal treatment or had any periapical lesions, a history of scaling and root planing, fractured teeth, or external resorption of the root or root caries. In this clinical trial, 120 patients diagnosed with severe chronic periodontitis were recruited, and a single hopeless tooth was extracted from each patient. The enrollment, allocation of patients and intervention given to the teeth is given in Fig. 9.

### Description of test instruments

The study used the following test curettes for subgingival instrumentation.

5/6 Gracey rigid curette (Hu-Friedy Co, Chicago, IL, USA).

5/6 After Five Rigid curette (Hu-Friedy Co, Chicago, IL, USA) whose terminal shank is elongated by 3 mm for access in deep periodontal pockets and blade is 10% thinner.

5/6 Mini Five Rigid curette (Hu-Friedy Co, Chicago, IL, USA) whose terminal shank is elongated by 3 mm and the blade is reduced to half-length of After Five or a standard Gracey curette and the blade that is 10% thinner than standard Gracey curette.

## Intervention

Using one of the test curettes, the lingual surface of each tooth was debrided. Each patient was randomly assigned to one of four groups. The computer-generated randomisation method (Random Function; Microsoft Excel, MS Office 360) assigned patients to a specific treatment modality group. A single operator performed subgingival instrumentation in vivo using a 5/6 Gracey Rigid Curette, a 5/6 After Five Rigid Gracey Curette, or a 5/6 Mini Five Rigid Gracey Curette. A total of 120 teeth were assigned equally to groups 1, 2, 3 and 4, respectively. Instrumentation was not performed in the control group and was titled group 1. The second group included patients that were instrumented with 5/6 Gracey Rigid curette. The third group of patients was instrumented with 5/6 After Five Rigid curette, while in the fourth group, 5/6 Mini Five Rigid curette was used as mentioned in Table 1.

**Table 1:** Participants Allocation and treatment assignments

Treatment arms	No. of subjects (N = 120)	Group titles	Treatment provided
Control	30	1	No Treatment
Gracey	30	2	Scaling by Gracey Curette
After 5	30	3	Scaling by After 5 Curette
Mini 5	30	4	Scaling by Mini 5 Curette

Local anesthesia was administered by infiltrating Lidocaine 1:100,000, and scaling and root planing were performed until a smooth and hard surface was detected by Explorer number 17. The level of the free gingival margin was marked with a small diamond round bur size 009 (DIA-Burs MANI) on a high-speed handpiece following instrumentation by curettes. This groove provided a landmark for evaluation under the scanning electron microscope. A control group was taken to compare the SEM photographs of instrumented test groups and non-instrumented control groups and to discern the difference between indentation marks produced by the curettes and the presence of smear layer before and after using the curettes.

### Examiner calibration

Instrumentation was performed in vivo by a single operator trained in periodontology with extensive experience, calibrated using a modified pen grasp technique for holding the curettes [38].

The angle between the cutting edge of the curette and the teeth was between 45 and 90 degrees, as well as a good finger rest allowed natural wrist-forearm motion. The shank of the instrument was kept parallel to the long axis of the tooth. The sharpness of the curettes was maintained by sharpening them with Arkansas stone every five strokes according to the manufacturer's instructions. (Hu-Friedy Co, Chicago, IL, US) [38]. The tooth was then extracted as atraumatically as possible, with the beak of the extraction forceps above the gingival margin. After extraction, the teeth were rinsed in running tap water for 60 s to remove all debris and blood and placed in a 0.9% NaCl solution to keep them hydrated until treatment.

### Preparation of root specimens for scanning electron microscopy

After instrumentation, the samples were fixed in Glutaraldehyde 2.5% in 0.1 M Phosphate Buffer, pH 7.4 for 24 h, and then washed thrice with phosphate buffer. All specimens were kept for 10 min in graded series of ethanol (50%, 70%, 85%, 96% ethanol) for dehydration [39]. They were dried overnight and then mounted on a 30 mm diameter aluminum stub with adhesive tape. The specimens were sputter-coated with a 300 Armstrong gold coating in the Auto coater.

### Scanning electron microscope analysis

**Fig. 1:**
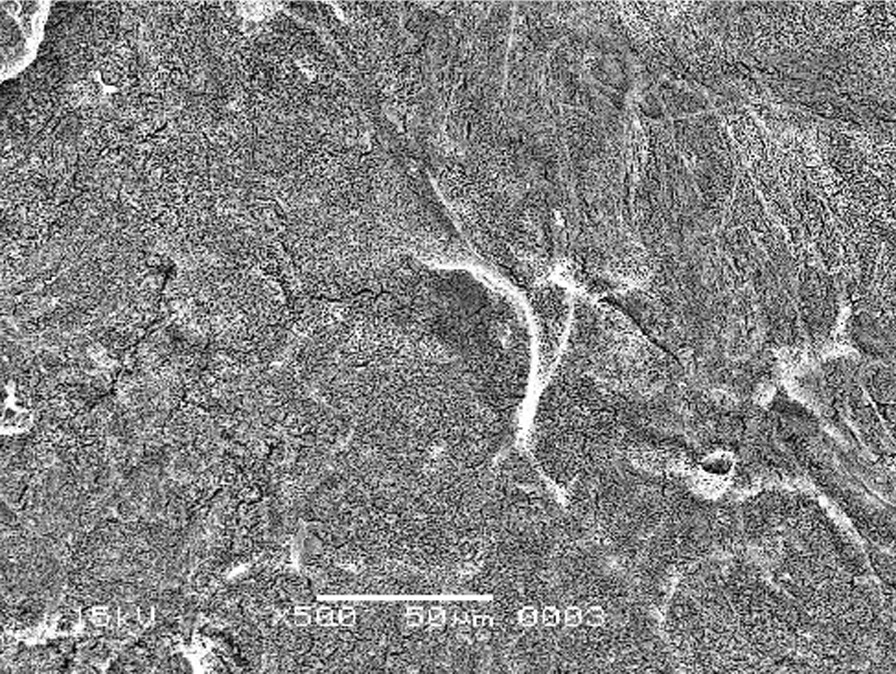
Grade 0 of root roughness index

**Fig. 2:**
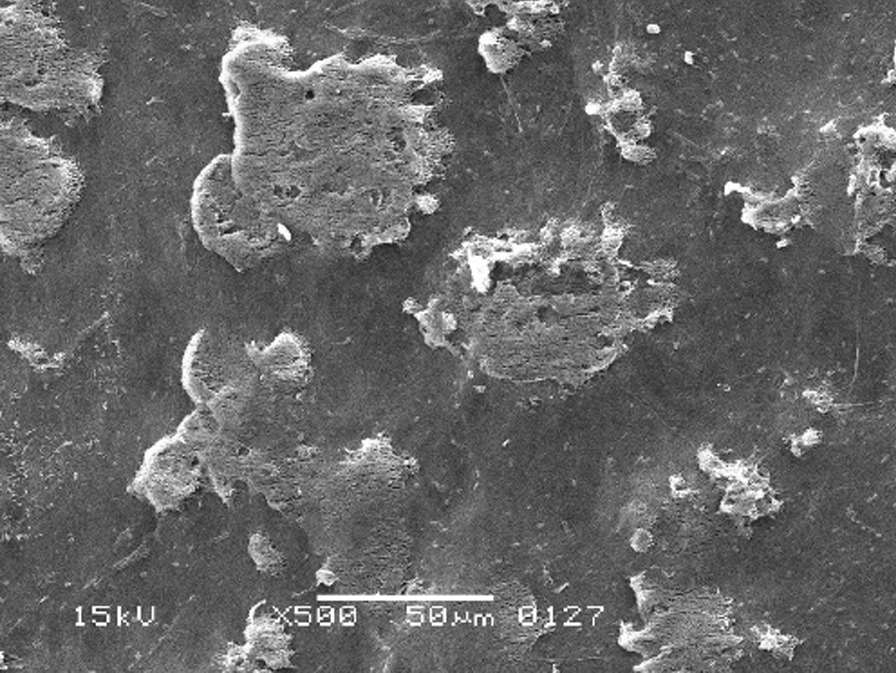
Grade 1 of root roughness index

**Fig. 3:**
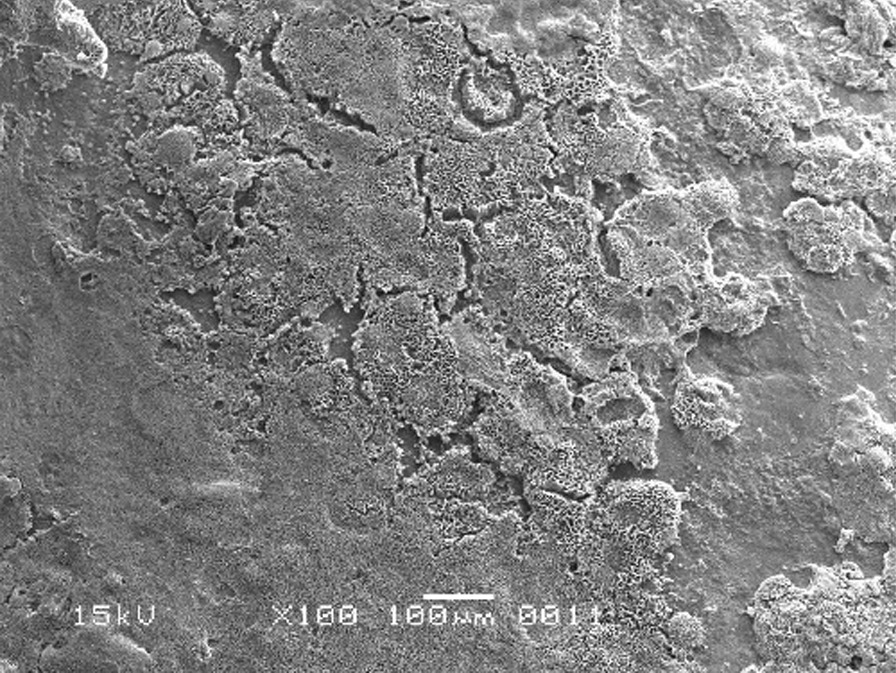
Grade 2 of root roughness index

**Fig. 4:**
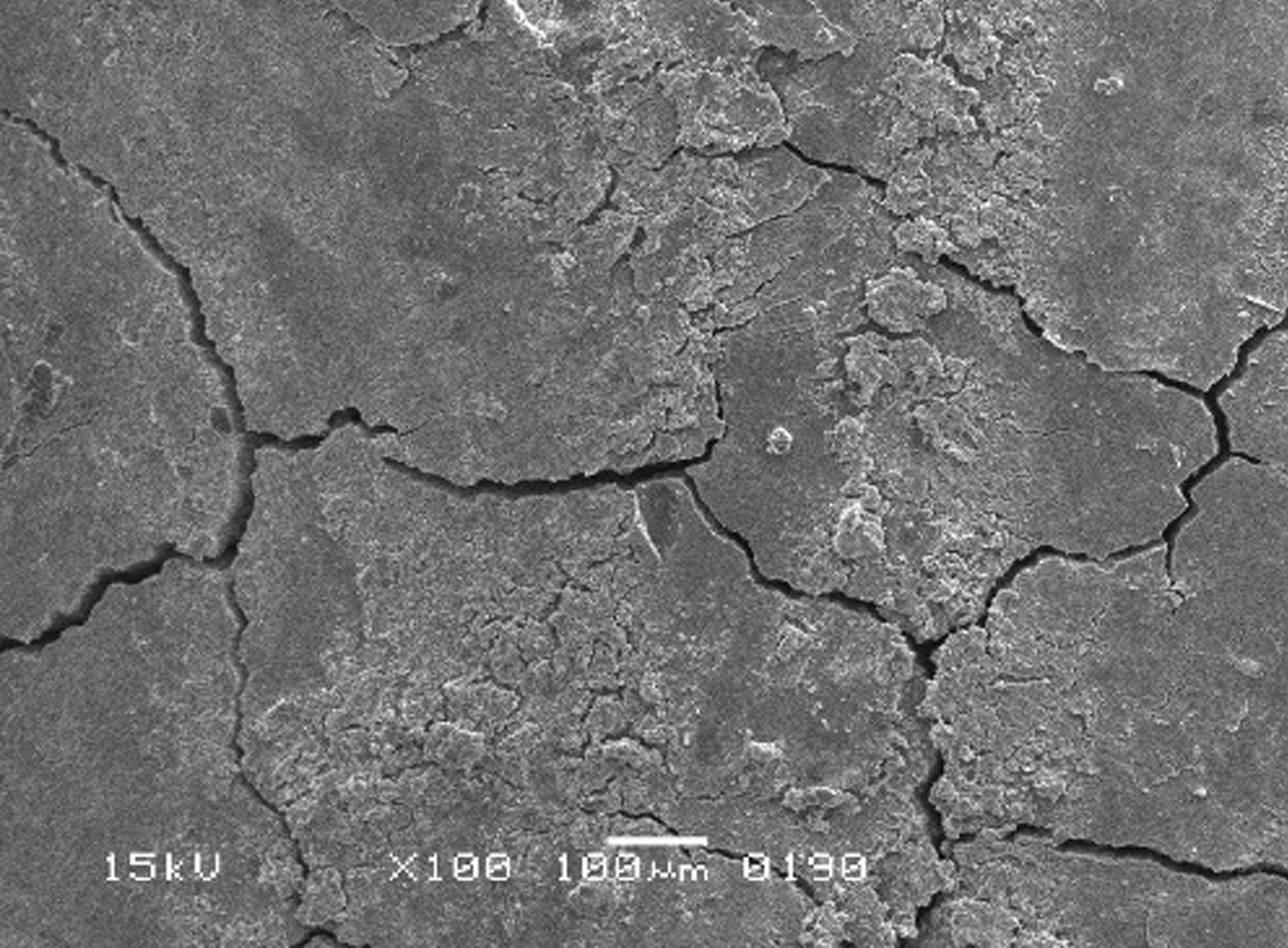
Grade 3 of the root roughness index

**Fig. 5:**
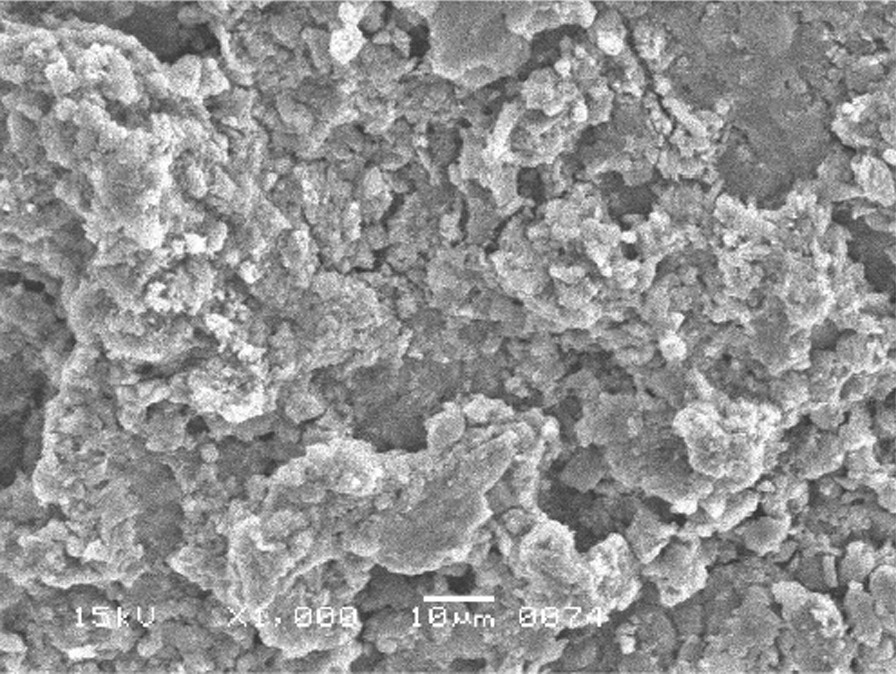
Grade 1 of smear layer index

**Fig. 6:**
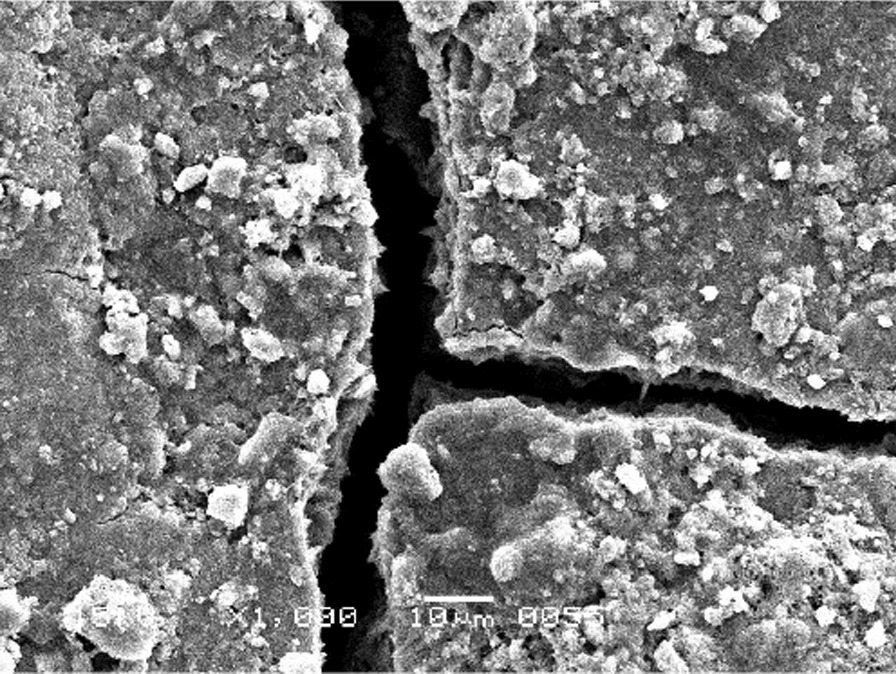
Grade 2 of smear layer index

**Fig. 7:**
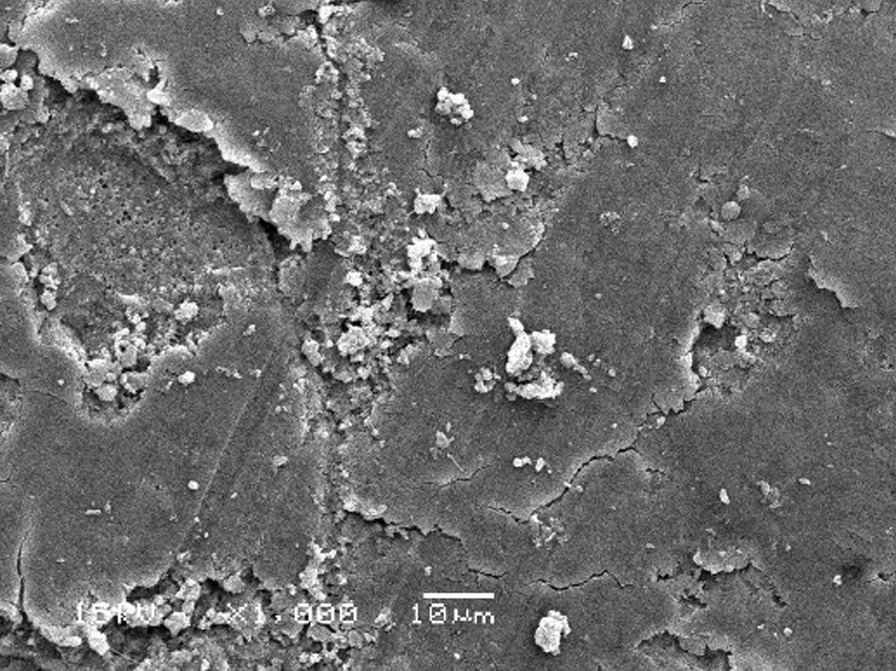
Grade 3 of smear layer index

**Fig. 8:**
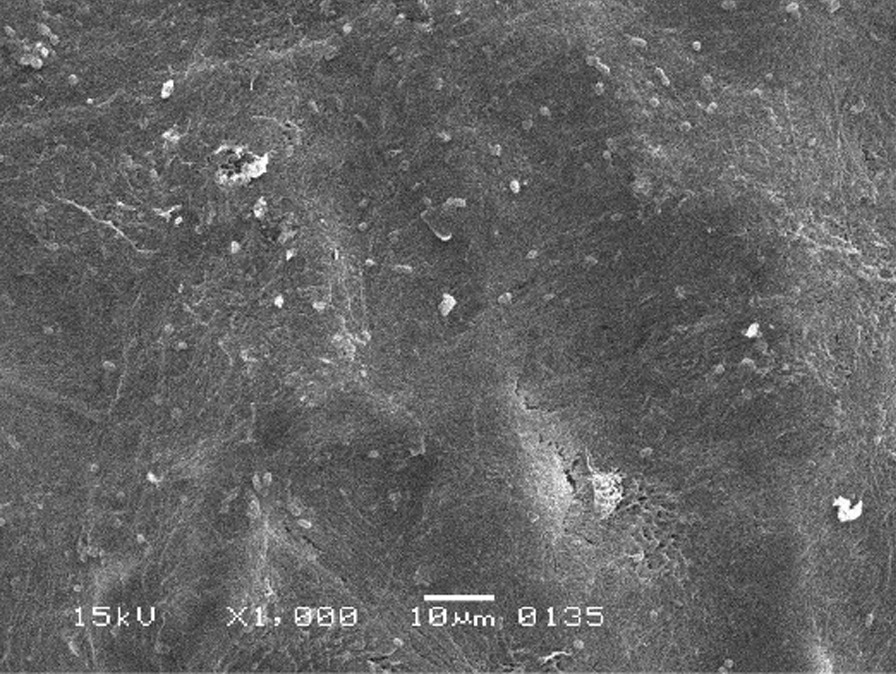
Grade 4 of smear layer index

The scanning electron micrographs were taken with SEM model no. JSM-6380A. Each specimen was blindly scored by two investigators. Six SEM photographs with different standardised magnifications were taken for each specimen which was later assessed using the indices "Roughness and Loss of Tooth Substance Index" [39] and "Smear Layer Index" [40], which were the primary outcomes. The “Roughness and Loss of Tooth Substance Index” [39] was analysed by taking the magnifications (× 100, × 200, and × 500) into consideration. The SEM representative photographs of the Root Roughness Index are given in Figs. 1, 2, 3 and 4. The “smear layer index" [40] was analysed using magnifications (× 1000, × 2000, and × 5000). The SEM representative photographs of the Smear layer Index are given in Figs. 5, 6, 7 and 8. For the computation of Root Roughness [39] and Smear layer Index [40], a single value was represented by taking the mode of the three readings taken on three different magnifications of both indices. The micrographs were assessed by two examiners blinded to the treatment groups. Inter-examiner reliability was checked to ensure the calibration of the examiners by applying the Intra-class correlation coefficient (ICC) test.

### Sample size calculation

The power of the test was calculated to justify the sample size of 120 samples using PASS version 15 software, based on a chi-square test with 95% confidence of interval, an effect size of 0.9146 with degrees of freedom 9 computed using results from an association between roughness index and instruments. It was found to be more than 99%. The same power of the test was found using results from an association between the smear layer index and instruments with an effect size of 0.854374 (9 degrees of freedom).

### Statistical analysis

The intraclass correlation method analyzed root roughness and smear layer formation for 30 teeth to determine inter-rater agreement. The ICC was found to be 0.91 for root roughness and smear layer, indicating strong agreement between the two examiners.

Data were analyzed using SPSS Version 21. Descriptive statistics were computed on patients' age, gender, brushing, and flossing habits. Similarly, descriptive statistics of teeth were made using frequencies and percentages. Pie charts were made using frequencies and bar charts using means.

**Fig. 9:**
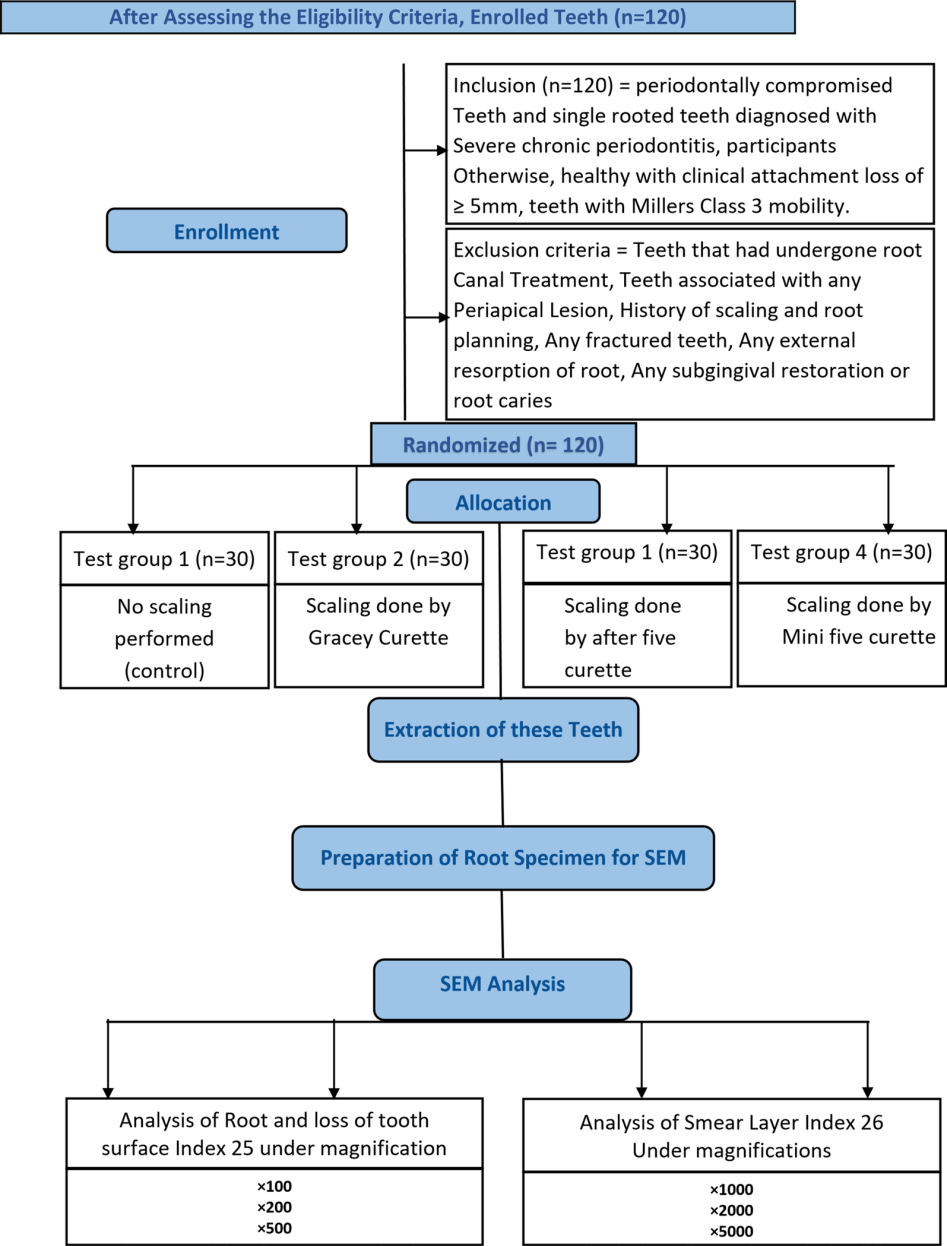
Study flow chart

Cross Tabulation was made between the test groups (Control, Gracey Curette, After five, and Mini Five) versus "Roughness and Loss of Tooth substance Index" [39]. Similarly, Cross Tabulation was made between the test groups versus the "Smear Layer Index" [40]. The Chi-square test with Bonferroni correction was used to determine how grades were distributed among groups. A p-value of 0.0031 or less was considered statistically significant (Fig. 9).

### Representative SEM photographs of root roughness index [39]

See Figs. 1, 2, 3 and 4.

### Representative SEM photographs of smear layer index [40]

See Figs. 5, 6, 7, 8 and 9.

## Results

### Demographic data

A total of 120 patients with 120 extracted teeth were recruited for this study. The average age of 56.5 years and standard deviation of 9.54 was present. Demographic data are summarised in Table 2.

**Table 2:** Descriptive analysis of patients

Characteristics	N = 120(%)
*Gender*	
Male	75 (62.5)
Female	45 (37.5)
*Age*	
35–45	19 (15.8)
46–55	43 (35.8)
56–65	32 (26.7)
66–75	26 (21.7)
*Brushing habit*	
No brushing	09 (7.5)
once daily	103 (85.8)
twice daily	08 (6.7)
*Floss*	
No	117 (97.5)
Yes	03 (2.5)

Table 2 represents the descriptive analysis of patients, showing the homogeneity of all groups based on their habits, age and gender distribution.

Table 3 represents the descriptive analysis of teeth showing the homogeneity of the group. Almost all of the participants had calculus index grade 3 and plaque index grade 1. The depth of periodontal pockets mostly ranged from 1 to 3 mm. These parameters demonstrate the homogeneity of the group based on calculus index, plaque index and depth of periodontal pockets

**Table 3:** Descriptive analysis of teeth

Characteristics	N = 120(%)
*Plaque index*	
Grade 0	18 (15)
Grade 1	66 (55)
Grade 2	36 (30)
Grade 3	00 (0)
*Calculus index*	
Grade 0	00 (0)
Grade 1	00 (0)
Grade 2	16 (13.3)
Grade 3	104 (86.7)
*PPD (BUCCAL)*	
1–3	94 (78.3)
4–6	21 (17.5)
≥ 7	05 (4.2)
*PPD (LINGUAL)*	
1–3	84 (70)
4–6	31 (25.8)
≥ 7	05 (4.2)
*BOP*	
No	56 (46.7)
Yes	64 (53.3)

Table 4 shows the comparison between Instruments and Roughness scores, showing a significant (p < 0.001) difference between the roughness scores among instruments, including control and the two curettes (Gracey and Mini-Five). The table also shows a comparison between test curettes and smear layer formation showing a significant (p < 0.001) difference between the smear layer scores among instruments, including control and the two curettes (After-Five and Mini-Five).

**Table 4:** Comparison between instruments and SEM analysis

SEM analysis	Instruments			
	Control n = 30 (%)	Gracey n = 30 (%)	After-five n = 30 (%)	Mini-five n = 30 (%)
*Roughness*				
Grade 0	5 (16.7)	0 (0)	0 (0)	0 (0)
Grade 1	22 (73.3)	1 (3.3)	04 (13.3)	0 (0)
Grade 2	3 (10.0)	17 (56.7)	05 (16.7)	07 (23.3)
Grade 3	0 (0)	12 (40.0)	21 (70.0)	23 (76.7)
Adjusted Residual	4.0	7.7	− 2.4	− 5.9
Adjusted P-value	< 0.001*	< 0.001*	0.017	< 0.001*
*Smear layer*				
Grade 1	0 (0)	11 (36.7)	22 (73.3)	24 (80.0)
Grade 2	6 (20.0)	15 (50.0)	06 (20.0)	05 (16.7)
Grade 3	10 (33.3)	4 (13.3)	02 (6.7)	01 (3.3)
Grade 4	14 (46.7)	0 (0)	0 (0)	0 (0)
Adjusted Residual	− 6.0	− 1.0	3.5	6.9
Adjusted P-value	< 0.001*	0.340	< 0.001*	< 0.001*

*Bonferroni correction (Significant at 0.05/16 = 0.0031)

**Fig. 10:**
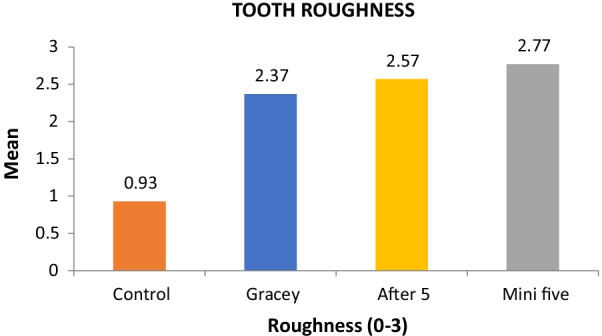
Comparison of the tooth roughness index of all test instruments with the control group. Roughness Index Means represented on Y-Axis. Test Instruments and Control group represented on X-Axis. Grade 0 RI—Smooth and even root surface without marks from instrumentation and with no loss of tooth substance. Grade 1 RI—Slightly roughened and corrugated local areas confined to cementum. Grade 2 RI—Definitely corrugated local areas where cementum may be removed entirely, although most of the overall cementum is still present. Grade 3 RI—Considerable loss of tooth substance with instrumentation marks into the dentin. The cementum is completely removed in large areas, or it has a considerable number of lesions from the instrumentation

Figure 10 shows the graphical bar representation of the Root Roughness Index among all the test curettes and the control group. In the control group, the roughness was very slight compared to the test groups. Among the three instruments, Gracey curette produced a smoother root surface compared to After Five and Mini Five curette, while Mini Five produced the maximum roughness, although there was not much significant difference between Mini Five and After Five curette.

**Fig. 11:**
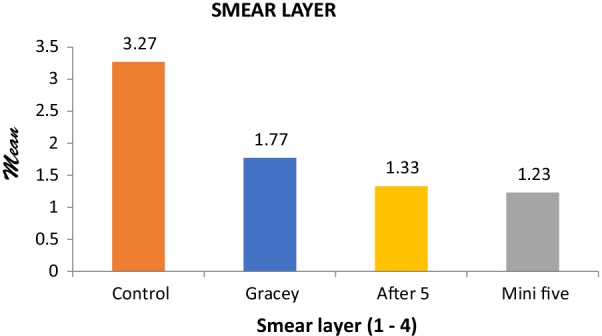
Comparison of Smear layer Index of all test instruments with the control group. Smear Layer Index Means represented on Y-Axis. Test Instruments and Control group represented on X-Axis. Grade 1 SLI—Thick and compact smear layer. Grade 2 SLI-Thin smear layer. Grade 3 SLI—Residues of smear debris. Grade 4 SLI—Absence of smear layer

Figure 11 shows the graphical bar representation of the Smear Layer Index among all the test curettes and the control group. Regarding smear layer formation, the control group showed minimum to almost absent smear layer.

Among the test instruments, Mini Five and After Five showed the maximum smear layer formation while the Gracey Curette showed less smear layer than the other two instruments.

### Scanning electron microscope descriptive analysis

When qualitative analysis of the surface appearance of the sample photographs was performed under a scanning electron microscope at different magnifications, it was observed that the control specimens were uneven and showed elevations and depressions due to the presence of foreign bodies, such as calculi, in the absence of scratches and gouging as shown in Fig. 13.

**Fig. 12:**
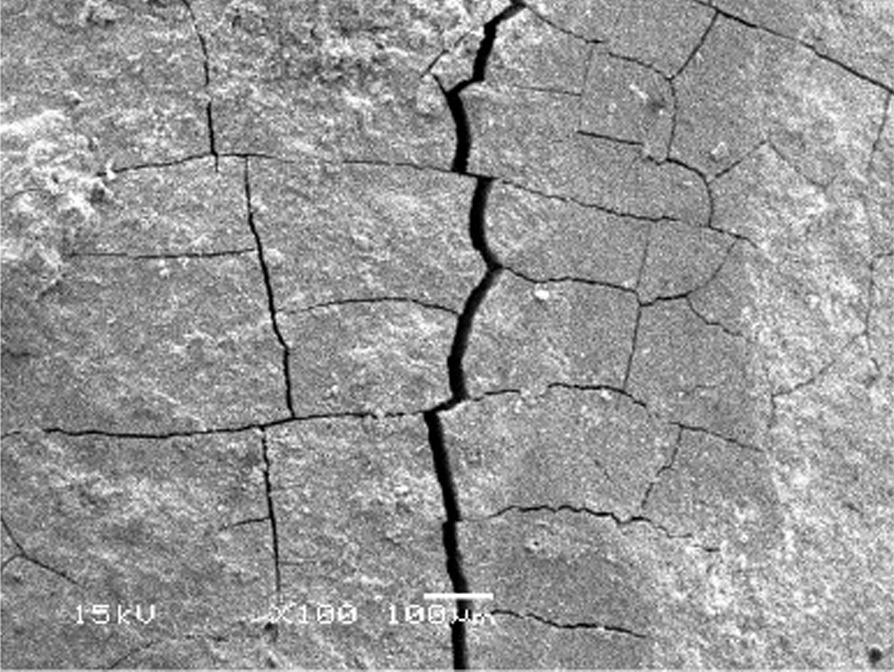
The crack pattern in instrumented group

**Fig. 13:**
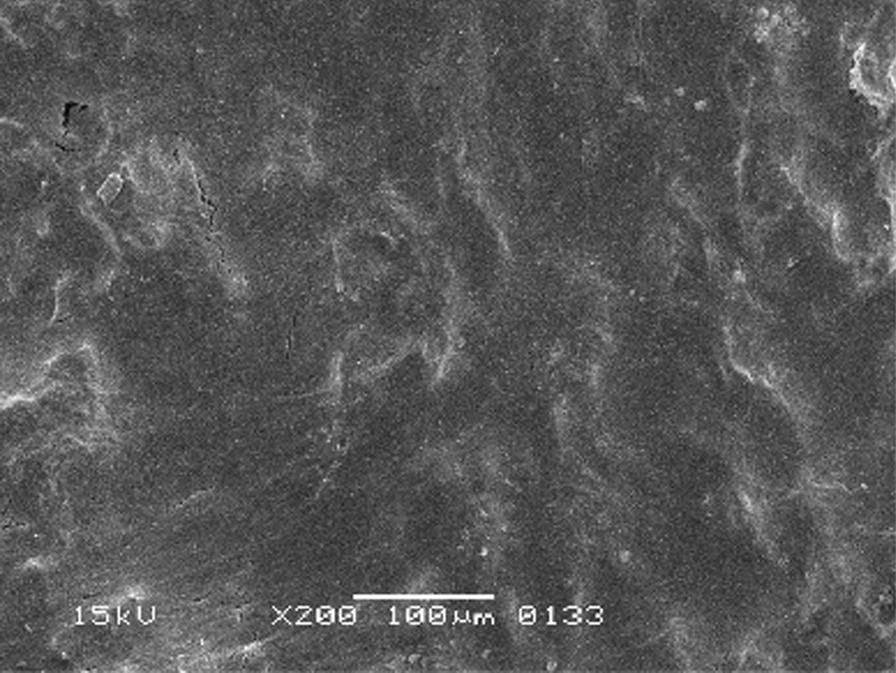
The control group

All instrumented groups showed deep scratches and gouging at various depths and diameters caused by hand instruments. Cracks ran in various horizontal and vertical directions, mostly making roughly box-shaped patterns and rough root textures, as shown in Figs. 12, 13.

## Discussion

It has been well established that there is no significant difference in periodontal pocket depth, clinical attachment levels, or bleeding upon probing when using ultrasonic instruments or manual instruments [41]. In contrast, there is a substantial difference in root roughness when using manual and ultrasonic instruments [27]. Regarding hand instruments, there is a paucity of studies that have compared the effects of debridement on root structure using separate hand instruments like Gracey, After Five, and Mini Five. In this study, the effects of the different curettes on root surface ultra morphology were examined in this study.

There were almost 62.5% males and 37.5% females in the present study. Studies have shown that males are more likely to develop periodontitis than females, suggesting gender differences [33].

There is scarce evidence comparing the effects of Gracey, After Five, and Mini Five curettes on root surfaces after debridement. In one study by Renato V.Alves, Gracey curettes were compared to Mini curettes (Mini Five) to evaluate the difference in trauma from instrumentation in terms of immediate attachment level before and after scaling. Renato concluded that root instrumentation causes an average trauma depth of 0.76 mm. No significant differences were found between the groups [42]. Matthew Andre conducted another study to compare the effects of different brands of Gracey curettes no 5/6, such as carbon steel and stainless steel, on root roughness and topography. He suggested that the quality of the cutting edge of the curette exerts a difference in the topography of the root surface. His study showed that stainless steel millennium had the most homogeneous root surface. In our study, in terms of smoothness, Gracey’s curette produced a smoother root surface compared to After Five and Mini Five Curettes, consistent with the study mentioned above [27].

Based on the results of the current study, which was an in vivo design and performed under SEM, we found that the Mini Five and After Five curettes produced greater root roughness than the Gracey curettes. These results were in accordance with the study by C.Landy. In vitro, he compared the long shank, short blade curette against the conventional Gracey curette variant (Curvette Sub-0) using a profilometer. He conducted the study on the maxillary and mandibular incisors set on the manikins and assessed the roughness of the root surface. His results verified that the test curette (Curvette-0) caused greater root roughness, although it was superior in removing the test material subgingivally [28].

In the present study, all the test groups that are Gracey, After Five, and Mini Five curettes, demonstrated some degree of gouging and cracks of varying sizes on the root surfaces. The root surfaces also exhibited deep scratches after root instrumentation. These results were congruent with many other studies that showed the presence of cracks and gouging with hand instruments [28, 43, 44].

Regarding smear layer production due to instrumentation with different curettes, the present study showed that the control group had almost absent or small residues of smear layer while all other instrument groups showed the presence of smear layer. These results correspond to one study by Priscilla Barbosa, in which root topography was analysed after different root treatments. In this study, the control specimens showed no smear layer, while the group scaled with Gracey curette showed the presence of a smear layer as observed in other studies [18]. However, in our study Gracey Curette showed a relatively less amount of smear layer compared to After Five and mini Five curettes. Mini Five and After Five showed a thick smear layer, while Gracey curette showed mostly a thin smear layer. These results are consistent with a study by Giles Gagnot in which two periodontists compared the root treatments using Gracey curettes and a mini-insert ultrasonic instrument under scanning electron microscopy. Compared to Periodontist B, fewer smear layers were produced on teeth treated by Periodontist A than on teeth damaged by scratching and cementum loss, with more smear layers shown by Periodontist B. A difference in cementum damage was caused by differences in lateral pressure exerted by the two periodontists [45].

Similarly, a recent clinical trial that evaluated clinical outcomes, chairside time, and post-treatment hypersensitivity using Gracey curettes, ultrasound and diamond burs revealed that Gracey curettes and ultrasound improved clinical performance attachment levels compared to diamond burs [46]. Our study provides the morphological basis for achieving better clinical outcomes with Gracey Curettes due to reduced root roughness and thin smear layer production. The results of this study are limited to single-rooted teeth only. While SEM is a convenient method for qualitative surface morphology analysis, profilometry is more suitable for quantitative analysis and could be used for a more comprehensive analysis of tooth root surfaces.

## Recommendations

In the future, more studies are needed to assess the ultra-morphology of root surfaces produced by different hand instruments, as there are few studies and inconsistent results. Different methodologies and techniques may be used to determine the results. More rigorous studies must be conducted because of the paucity of research on long-shank curettes.

## Conclusion

Gracey curettes produced a relatively smoother root surface with less smear layer formation than After Five and Mini Five curettes, which produced a relatively roughened root surface with a thicker smear layer. There was no difference between Mini Five and After Five in root roughness and smear layer. Differential results may be caused by the sharpness of instrument blades and variations in the diameter of the blade and shank length. Studies are needed in this area to determine the impact of these new curettes on root morphology.

## Data Availability

The data sets analysed during the current study are available from the corresponding author on reasonable request.

## Abbreviation

SEM: Scanning electron microscope.
